# The impact of intraoperative hemoadsorption during cardiac surgery for infective endocarditis

**DOI:** 10.1186/s40635-026-00988-0

**Published:** 2026-10-14

**Authors:** Amin Serradj, Gabriel Saiydoun, Costin Radu, Eric Bergoend, Antonio Fiore, Raphaelle Huguet, Pascal Lim, Cyrus Moini, Thierry Folliguet

**Affiliations:** 1 Chirurgie Cardiaque et Transplantation, Assistance-Publique Hôpital Henri Mondor, Université Paris 12 UPEC, Paris, France; 2 Service de Cardiologie, Assistance Publique Hôpital Henri Mondor, Université Paris 12 UPEC, Paris, France; 3 Service de Cardiologie, Hôpital de Melun, Melun, France; 4 Department of Thoracic and Cardiovascular Surgery, Henri Mondor University Hospital APHP, 1 rue Gustave Eiffel, Créteil, 94000 Paris France

**Keywords:** Infective endocarditis, Hemoadsorption, Cytokines, Mortality, Cardiac surgery, CytoSorb vasoplegia

## Abstract

**Background:**

Postoperative sepsis and/or systemic inflammatory response syndrome caused by infective endocarditis (IE) can lead to significant postoperative mortality. Intraoperative hemoadsorption therapy has shown promising results in the treatment of sepsis and dysregulated inflammatory response during cardiac surgery. The aim of this study was to evaluate the effects of intraoperative hemoadsorption in high-risk patients with IE undergoing cardiac surgery.

**Methods:**

A total of 176 high-risk patients with IE were enrolled from 10/2020 to 10/2024. Patients with intraoperative hemoadsorption (HA group, *n* = 93) were compared to patients without hemoadsorption (control, *n* = 83). The primary endpoint was a composite of 30-day mortality, incidence of vasoplegic syndrome within the first 24 h and 7 days postoperatively, and intensive care (ICU) stay, analyzed using an unmatched hierarchical win-ratio method.

**Results:**

There were no differences in baseline characteristics between the groups. The mean hemoadsorption time was 106 ± 61 min in the HA group. Vasoplegic syndrome was more frequent in the control group (49.4% vs. 24.7%, *p* < 0.001). Importantly, 30-day mortality was also significantly lower with intraoperative hemoadsorption (7.5% vs. 20.5%, *p* = 0.003). The mean ICU stay was shorter in the HA group (7.8 ± 4.6 vs. 11.5 ± 4.2 days, *p* < 0.001). The analysis met the primary endpoint in favor of patients receiving intraoperative hemoadsorption (win ratio 3.83; 95% CI 2.55, 5.74; *p* < 0.001).

**Conclusions:**

In high-risk cardiac surgery patients with IE, the intraoperative use of hemoadsorption was associated with a significantly lower incidence of postoperative vasoplegic syndrome and lower overall 30-day mortality. These findings should be interpreted in the context of the study’s methodological limitations and confirmed in future randomized controlled trials.

**Trial registration:**

This study was approved by the Institutional Review Board (IRB00012919) of the French Society of Thoracic and Cardiovascular Surgery (CERCSFCTCV20251021).

**Supplementary Information:**

The online version contains supplementary material available at https://doi.org/10.1186/s40635-026-00988-0.

## Background

The incidence of fulminant infective endocarditis (IE) has risen alarmingly annually in all countries over the last decade. This trend may be exacerbated by the increasing number of prosthetic heart valves being implanted. Of note, staphylococci and streptococci were the most common pathogens identified [1, 2]. Many of these patients require early treatment despite being on anti-infective therapy, due to hemodynamic compromise. The rationale for early therapy is infection control (especially in “hot” IE), and prevention of both progressive heart failure with irreversible structural damage, and systemic embolism [3]. However, mortality remains high, and the most feared post-operative complication remains refractory hemodynamic instability, with vasoplegia being one of the main mechanisms [4]. This systemic inflammatory response in combination with systemic infection results in severe sepsis orchestrated by various cytokines as messengers [5]. Vasoplegia adversely affects outcomes in patients with an urgent surgical indication due to uncontrolled infection, disease progression, or hemodynamic deterioration [4]. Intraoperative hemoadsorption therapy has shown promising results in the treatment of sepsis and dysregulated inflammatory response during cardiac surgery [6, 7]. Similarly, it has been previously reported that intraoperative hemoadsorption could potentially reduce the incidence of postoperative sepsis and sepsis-related death in patients presenting with IE [8].

Therefore, in the present study we sought to evaluate the impact of intraoperative hemoadsorption therapy in high-risk patients presenting with proven infective endocarditis undergoing cardiac surgery.

## Methods

### Patients

The present study was a non-randomized, single-center prospective study. Between September 2022 and October 2024, 193 consecutive high-risk patients underwent cardiac surgery due to IE at the Department of Cardiac Surgery, Henri Mondor University Hospital, Creteil, Paris. All eligible participants were patients with definite infective endocarditis who underwent surgical therapy with cardiopulmonary bypass (CPB). For the present analysis, the modified Duke criteria were used to verify definite IE (2 major criteria, 5 minor criteria, or 1 major + 3 minor criteria). All patients were discussed within the institutional multidisciplinary endocarditis team.

Exclusion criteria were: [1] cardiac device-related IE, requiring simple retraction without CPB [2], IE of a transcatheter aortic valve (TAVI) or [3] patients with incomplete follow-up. For each individual patient, the EuroSCORE-II (European System for Cardiac Operative Risk Evaluation) was calculated using the online calculator (https://www.euroscore.org/). The present study was performed according to the Declaration of Helsinki and has been reported in line with the STROCSS *(Strengthening the Reporting of Cohort*,* Cross-Sectional and Case-Control Studies in Surgery)* criteria. This study was approved by the Institutional Review Board (IRB00012919) of the French Society of Thoracic and Cardiovascular Surgery (CERC-SFCTCV-2025–10–21). Consent was obtained either from the patient or, if the patient lacks capacity to provide consent, from a legally authorized representative. Two dedicated research nurses routinely enter patients undergoing cardiac surgery at Henri Mondor Hospital into a national database called EPICARD. The hospital is a designated tertiary referral center for endocarditis in the greater Paris area, with referrals made via a dedicated telephone number and email address, allowing cases to be discussed at any time. Since the endocarditis team was created in 2015, 1,269 patients have been referred to our center. All cases are discussed and reviewed by the dedicated staff team every week. All patients referred to the center are entered into an endocarditis database by the research nurses, and follow-up is conducted by the infectious team at the center in the first year, at three, six and twelve months. Blood cultures analysis cardiac echography and or CT scan are performed at each visit. Other exams are performed at demand depending on the patient condition and evolution. After 1 year the patient is followed by a cardiologist.

Following the exclusion of 17 patients due to incomplete or lost follow-up data, 176 patients were eligible for inclusion in our study. We compared the results from two years before and two years after we introduced intraoperative hemoadsorption to our center. Hemoadsorption therapy with CytoSorb was introduced in September 2022. Between September 2022 and October 2024, 93 consecutive patients underwent surgery using intraoperative hemoadsorption (CytoSorb group). This group was then compared with 83 consecutive patients who underwent surgery without hemoadsorption between January 2020 and September 2022 (control group). Apart from the use of hemoadsorption, no other modifications to the surgical team or perioperative management were performed, with the same surgeons and anesthetic team involved.

### Outcome measures

The primary endpoint was a composite of 30-day mortality, the incidence of vasoplegic syndrome within the first 24 h and additionally on postoperative day 7 (as defined as low blood pressure despite optimal fluid overload >2 L of saline perfusion, requiring vasopressor therapy (norepinephrine ≥ 0.5 mg/h) to maintain a mean arterial pressure (MAP) > 65 mmHg despite adequate fluid resuscitation, in the presence of preserved left ventricular systolic function (LVEF > 45%), with or without inotropic support) and ICU-stay analyzed by an unmatched hierarchical win ratio method. In addition, there should be no evidence of sepsis by positive blood culture, hemorrhagic shock or pericardial tamponade as an underlying cause of vasoplegia.

Survival was determined by active follow-up at 90 days (consultation or by national electronic health identification number). Secondary endpoints included postoperative need for mechanical circulatory support (intra-aortic balloon pump - IABP or extracorporeal membrane oxygenation - ECMO), dialysis or reintubation, and hospital stay. Chronic obstructive pulmonary disease (COPD) was defined according to the EuroSCORE definition. Patient and operative demographics were recorded in a prospective institutional database and extracted and analyzed retrospectively.

### Hemoadsorption therapy

**Fig. 1:**
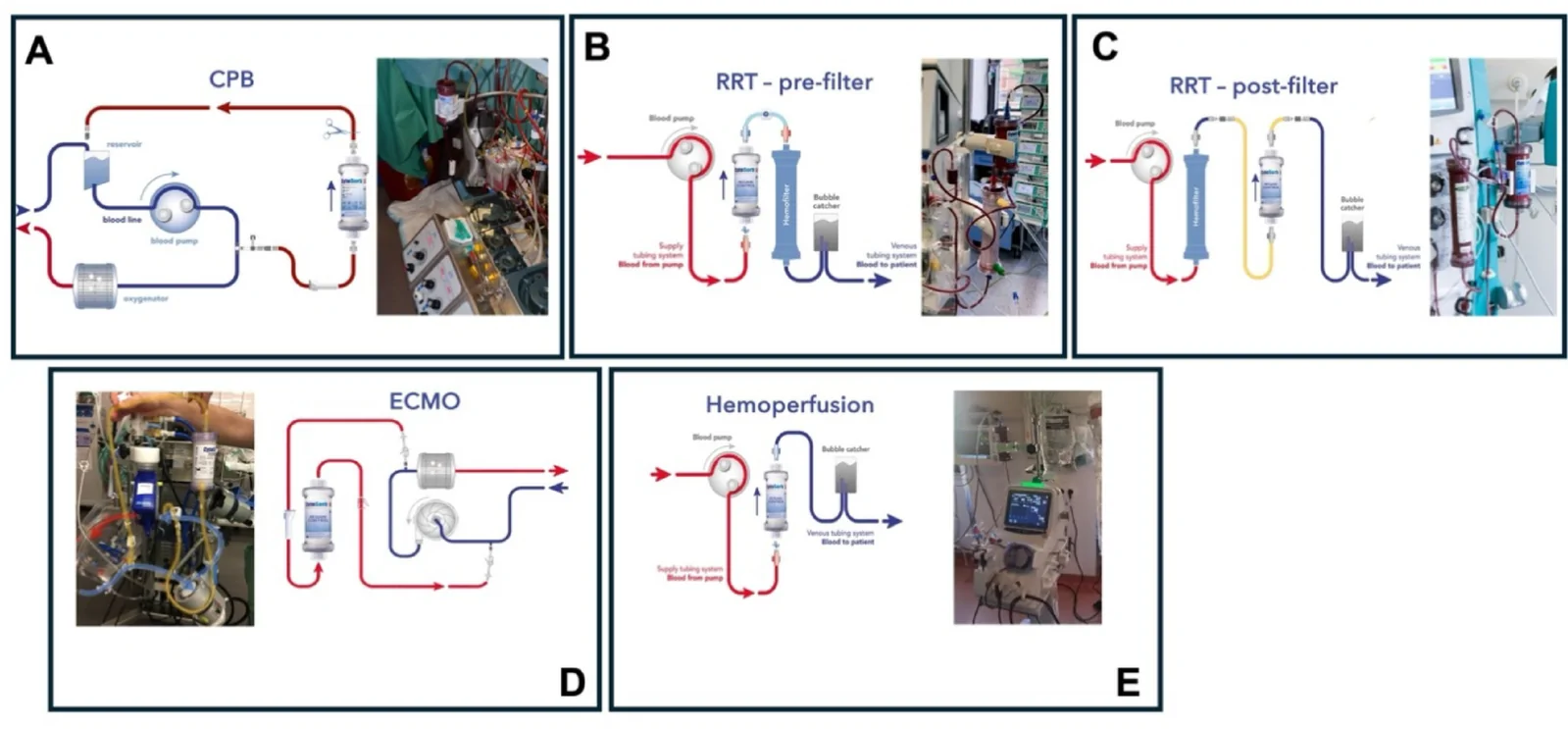
Potential implantation modes of hemoadsorption (CytoSorb) in various extracorporeal circulation circuits (**A**: Cardio-Pulmonary Bypass (heart-lung machine), **B**: Renal Replacement Therapy (RRT) dialysis (pre (**B**) and post filter (**C**)), **D**: Extracorporeal Membrane Oxygenation (ECMO), E: hemoperfusion)

The concept of hemoadsorption therapy with CytoSorb is based on extracorporeal blood purification. Hemoadsorption effectively reduces excessive levels of various inflammatory mediators with the aim of specifically modulating the exaggerated immune response, mitigating the cytotoxic effects of elevated cytokine levels and increasing the chances of recovery. The CytoSorb hemoadsorption cartridge is filled with highly biocompatible unique porous polymer beads coated with divinylbenzene. Each polymer bead is between 300 and 800 μm in size and has multiple pores and channels, resulting in an effective surface area of approximately 40,000 m^2^, effective for hydrophobic binding of small and medium-sized molecules up to 60 kD, including cytokines. The cartridge can be safely and easily integrated into various extracorporeal circuits, such as e.g., continuous renal replacement therapy, heart-lung machines and ECMO (Fig. 1).

The CytoSorb adsorber is CE (Conformité Européenne) approved and allows removal of cytokines and other inflammatory mediators. Moreover, other substances such as free hemoglobin, myoglobin, bilirubin and bile acids, bioactive lipids, light chains of antibodies, toxic metabolites, bacterial exotoxins (toxins such as shiga, alpha-hemolysin, gas gangrene, diphtheria) and other toxins can be removed by the adsorber. In addition, CytoSorb is indicated for the removal of the P2Y_12_ inhibitor ticagrelor and/or the factor Xa inhibitor rivaroxaban intraoperatively during surgical procedures involving cardiopulmonary bypass.

### Postoperative care

All patients were admitted to the cardiac intensive care unit (ICU) after surgery with invasive hemodynamic monitoring and guideline-directed antibiotic and supportive therapy. Hemodynamic monitoring included standard arterial and central venous catheterization for invasive measurement of blood pressure and oxygen saturation. Fluid and vasopressor therapy (mainly epinephrine and norepinephrine) was guided by several factors, including blood pressure, heart rhythm, preoperative cardiac function, and metabolic parameters. Our standardized institutional protocol for vasopressor management was used throughout the study period. Norepinephrine was the first-line vasopressor, with dose escalation and the addition of second-line vasopressors (e.g., vasopressin) at the discretion of the treating intensivist according to predefined hemodynamic targets and clinical response. All cases of vasoplegic syndrome were identified prospectively during routine postoperative care using these standardized criteria. In accordance with this protocol, adequate volume status was defined as a central venous pressure (CVP) of 12–14 mmHg after the administration of at least 2 L of crystalloid solution during the immediate postoperative period (within the first 24 h).

### Operation techniques

Surgery was performed under general anesthesia and endotracheal intubation. Preoperative transesophageal echocardiography was performed to assess cardiac and valvular function. Standard aortic and caval (or bicaval) cannulation techniques were used. Cardioplegic arrest was achieved using crystalloid Bretschneider cardioplegia (Custodiol, Dr Franz Koehler Chemie, Bensheim, Germany) or warm blood cardioplegia (Calafiore) according to surgeon preference. For cases selected for intra-operative hemoadsorption, a hemoadsorption device Cytosorb (Cytosorbents^®^, Princeton, NJ, USA) was placed in a parallel circuit of the heart-lung machine during cardiac surgery.

### Statistical analysis

Data was analyzed using SPSS software version 29 (IBM, Armonk, NY, USA) and the SAS software package version 9.4M8 (SAS Institute, Cary, NC, USA). Continuous variables were expressed as mean and median with standard deviation (SD) and interquartile range (IQR), respectively, and compared using Student’s t-test or the Mann-Whitney test. Categorical data were expressed as number of patients and frequencies and compared using the chi-square test. Survival curves were generated with the Kaplan-Meier method. A *p* value of less than 0.05 was considered to indicate statistical significance. The primary composite effectiveness endpoint was evaluated using the win ratio method of analysis for a hierarchical composite endpoint based on the Finkelstein- Schoenfeld test with 3 components ranked in order of clinical importance: [1] 30-day mortality; [2] incidence of postoperative vasoplegic syndrome; and [3] ICU-stay [9, 10]. The supplemental composite effectiveness endpoint included [1] 30-day mortality; [2] incidence of postoperative vasoplegic syndrome; [3] ICU-stay and [4] acute kidney injury. The win ratio assigns “wins,” “losses”, or “ties” in sequential comparisons between treatment and control arm subjects according to the hierarchical order of the components [10]. A win ratio greater than 1.0 favors the treatment arm; when the lower bound of a 95% confidence interval (CI) around the ratio exceeds 1.0, the treatment is shown to be statistically superior to the control at alpha = 0.05.

## Results

### Baseline characteristics

Between January 2020 and October 2024, 176 consecutive patients underwent cardiac surgery with cardiopulmonary bypass for infective endocarditis, with the introduction of intraoperative hemoadsorption in September 2022. All patients were classified as high-risk patients with proven endocarditis. Overall, the patients had a high-risk profile, with a mean EuroSCORE-II of approximately 12% in both groups. All patients had an urgent indication for surgery and almost half of the patients in both groups were in NYHA (New York Heart Association) functional class III or IV. Almost a fifth of both groups were redo cases with previous cardiac surgery. Ninety-three consecutive patients operated with hemoadsorption after September 2022 were compared to 83 patients with IE operated before September 2022 without hemoadsorption.

Surgical and anesthesiologist team was the same during the entire period, and no procedural changes were performed except adding a CytoSorb cartridge on the CPB machine.

There was an almost equal distribution of age at the time of surgery between the two groups (60.4 (81 − 21) vs. 61.4 (87 − 24) years (*p* = 1.00) and most patients in both groups were male. The median time between definitive diagnosis of infective endocarditis and surgery was 13 days versus 9 days (*p* = 0.69) in the hemoadsorption and control group. Prior to surgery, 11 patients in the hemoadsorption group and 9 patients in the control group were ventilated. In addition, a total of 8 patients in the hemoadsorption group and 9 patients in the control group received preoperative vasopressor therapy. There were no significant differences in other demographics, hemodynamic and pulmonary status, and levels of preoperative inflammatory parameters between the two groups. The preoperative baseline characteristics of the patients are shown in Table 1.

**Table 1:** Baseline characteristics

Variable	CytoSorb group *n* = 93	Control group *n* = 83	*p*-value
**Demographics**			
Age, years	60.4 ± 14.2	61.4 ± 13.9	1.00
Gender, male	73 (78.5)	64 (77.1)	0.72
BMI, kg/m^2^	26.5 ± 3.0	26.2 ± 2.5	1.00
Systemic hypertension	51 (54.8)	48 (57.8)	0.76
Time between diagnosis and surgery, days	13 (6–20)	9 (4–15)	0.69
Coronary artery disease	16 (17.2)	21 (25.3)	0.20
COPD	4 (4.3)	3 (3.6)	1.00
Diabetes	17 (18.3)	18 (21.7)	0.57
Dialysis dependent	6 (6.4)	8 (9.6)	0.58
Liver disease	1 (1.1)	3 (3.6)	0.34
Peripheral vascular disease	19 (20.4)	16 (19.3)	1.00
Atrial fibrillation	13 (14.0)	11 (13.2)	1.00
Re-Operation	21 (22.6)	19 (22.9)	1.00
LV-ejection fraction, %	59 ± 10	61 ± 11	0.94
Preoperative stroke	1 (1.1)	2 (2.4)	0.60
Fever (≥ 38.5 C°) %	58 (62.3)	52 (62.6)	1.00
Hemoglobin preoperatively, g/dL	9.8 ± 1.2	9.9 ± 1.1	1.00
Urgent indication	93 (100.0)	83 (100.0)	1.00
EuroSCORE II, %	13 ± 8	12 ± 7	0.99
NYHA class III/IV	42 (45.2)	39 (47.0)	0.88
CRP, mg/dL	9.3 ± 7.3	8.7 ± 6.4	0.83
**Clinical status**			
Intubated	11 (11.8)	9 (10.4)	1.00
Vasopressor need	8 (8.6)	9 (10.8)	0.62

Data are presented as mean±SD (standard deviation), number (%) or median (min.-max.), BMI, Body mass index; EuroSCORE, European System for Cardiac Operative Risk Evaluation; COPD, Chronic Obstructive Pulmonary Disease., CRP, C-Reactive Protein., NYHA New York Heart Association, LV, Left Ventricle

### Operative characteristics

Table 2 shows the operative characteristics of the two groups. Infective endocarditis involved multiple valves. The majority of patients in both groups presented with IE of the aortic valve. Mitral valve IE was present in 29 patients in the hemoadsorption group and 33 patients in the control group. Prosthetic valve infective endocarditis occurred in a total of 29 patients, 16 in the hemoadsorption group and 13 in the control group. Concomitant coronary artery bypass grafting was performed in 4.3% of patients in the hemoadsorption group compared to 3.6% of patients in the control group. Both cardiopulmonary bypass (CPB) and aortic cross-clamp times were comparable between the two groups with slightly longer CPB-times in controls (p = ns). Both groups did not differ regarding the volume of cardioplegia administered intraoperatively (*p* = 0.49). The complexity of the surgical procedures did not differ significantly between the two groups.

**Table 2:** Operative characteristics

Variable	CytoSorb group *n* = 93	Control group *n* = 83	*p*-value
Mitral valve surgery	29 (31.2)	33 (39.8)	0.27
Isolated mitral valve surgery	32 (34.4)	24 (28.9)	0.43
Mitral valve repair	2 (2.1)	4 (4.8)	0.33
Aortic valve surgery	56 (60.2)	48 (57.8)	0.76
Isolated Aortic valve surgery	39 (41.9)	38 (45.8)	0.26
Commando procedure	3 (3.2)	0 (0)	0.10
Tricuspid valve surgery	8 (8.6)	11 (13.2)	0.34
Prosthetic valve endocarditis	16 (17.2)	13 (15.7)	0.84
Concomitant CABG	4 (4.3)	3 (3.6)	1.00
Cardiopulmonary bypass time, minutes	122 ± 58	149 ± 75	0.53
Intraoperative CytoSorb use, minutes	106 ± 51	---	
Aortic cross-clamp time, minutes	99 ± 48	104 ± 10.7	0.72
Skin-to-skin time, hours	4.5 ± 1.5	4.3 ± 1.4	0.77
Post-CPB vena cava inferior dilatation	10 (10.8)	5 (6.0)	0.26

Data are presented as number (%); CABG, coronary artery bypass grafting

### Endpoints

**Fig. 2:**
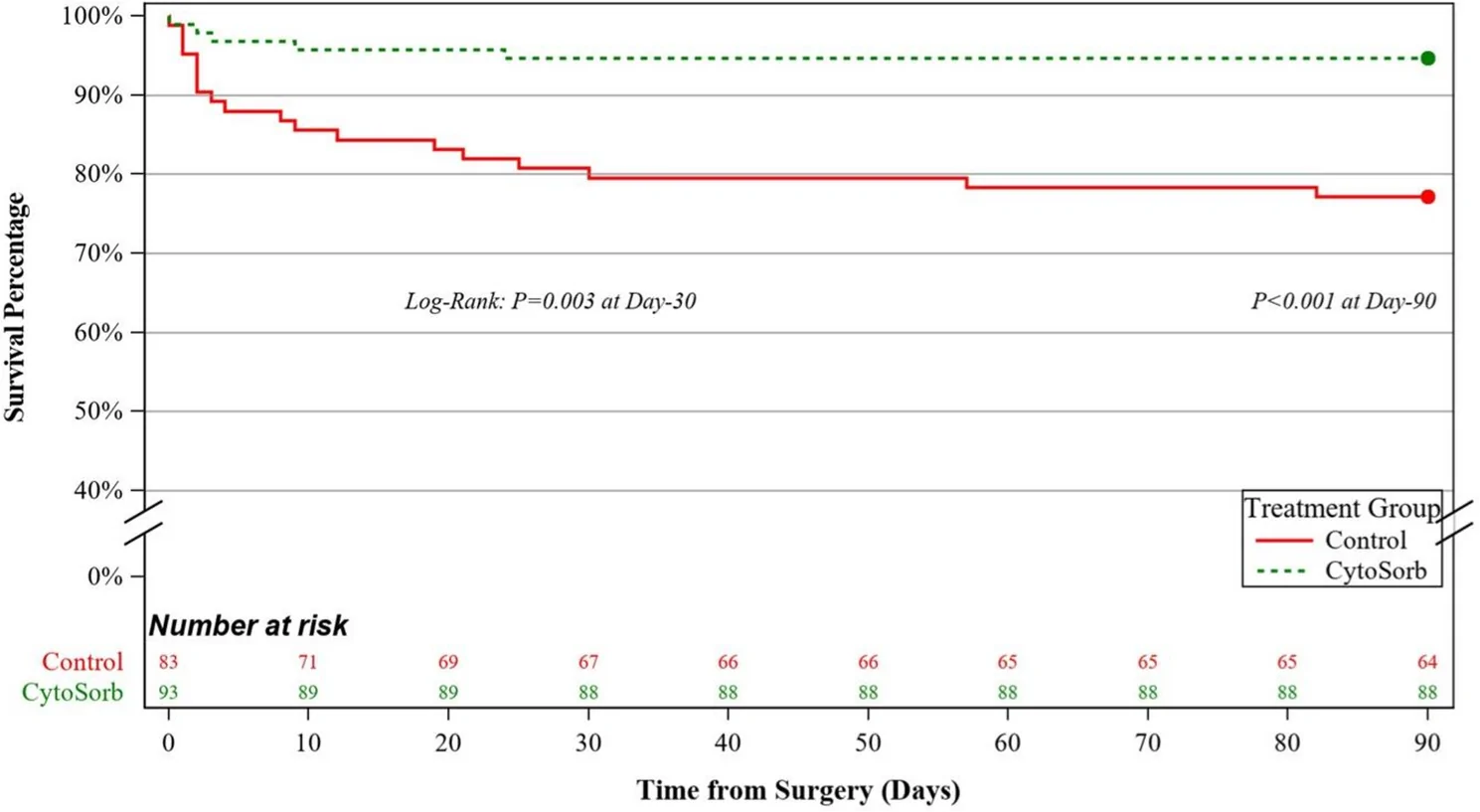
Kaplan-Meier survival curve up to 90 days. Intraoperative hemoadsorption with CytoSorb (green dotted line), control (red line)

Mortality at 30 days was 7.5% in the hemoadsorption group (7/93) compared to 20.5% in the control group (17/83); HR 0.33; 95%CI: 0.14, 0.78, *p* = 0.008). Similarly at 90 days, mortality in the hemoadsorption group was significantly lower (8/93) compared to controls (19/83: HR 0.35; 95%CI: 0.15, 0.80, *p* = 0.009). Vasoplegic syndrome within the first 7 days postoperatively occurred more frequently in the control group (*p* < 0.001). Of note, all cases of vasoplegic syndrome occurred within the first 24 h postoperatively. ICU stay was shorter in the hemoadsorption group, (7.8 ± 4.6 vs. 11.5 ± 4.2 days, *p* < 0.001). The endpoints are summarized in Table 3. The Kaplan Meier survival curve is presented in Fig. 2.

**Table 3:** Endpoints

Variable	CytoSorb group *n* = 93	Control group *n* = 83	*p*-value	
**Primary**				
30-day mortality	7 (7.5)	17 (20.4)	0.003	
90-day mortality	8 (8.6)	19 (23.1)	0.009	
Vasoplegic syndrome	23 (24.7)	41 (49.4)	< 0.001	
ICU-stay, days	7.8 ± 4.6	11.5 ± 4.2	< 0.001	
**Secondary endpoints**				
Postoperative IABP/ECMO	4 (4.3)	6 (7.2)	0.52	
Dialysis	13 (14.0)	21 (25.3)	0.08	
Reintubation	7 (7.5)	5 (6.0)	0.77	
Hospital-stay, days	15.3 ± 13.7	18.9 ± 15.7	0.18	
**Additional endpoints**				
Pneumonia	6 (6.5)	9 (10.8)	0.42	
Prolonged ventilation (> 24 h)	6 (6.5)	9 (10.8)	0.42	
Hemoglobin within first 24 h, g/dL	8.6 ± 0.9	8.5 ± 0.8	1.00	
Patients receiving RBC transfusion within 24 h	54 (58.1)	45 (54.2)	0.61	
Patients receiving FFPs within 24 h	34 (36.6)	22 (26.5)	0.15	
Patients receiving platelet transfusion within 24 h	31 (33.3)	27 (32.5)	0.91	
Patients receiving coagulation factors (e.g. fibrinogen or prothrombin complex)	16 (17.2)	10 (12.0)	0.34	
Surgical revision	11 (11.8)	9 (10.8)	0.83	

Data are presented as number (%) or median (IQR) IABP, intra-aortic balloon-pump; ECMO, extracorporeal membrane oxygenation; ICU, intensive care unit; RBC, Red blood cell; FFP, Fresh frozen plasma

**Fig. 3:**
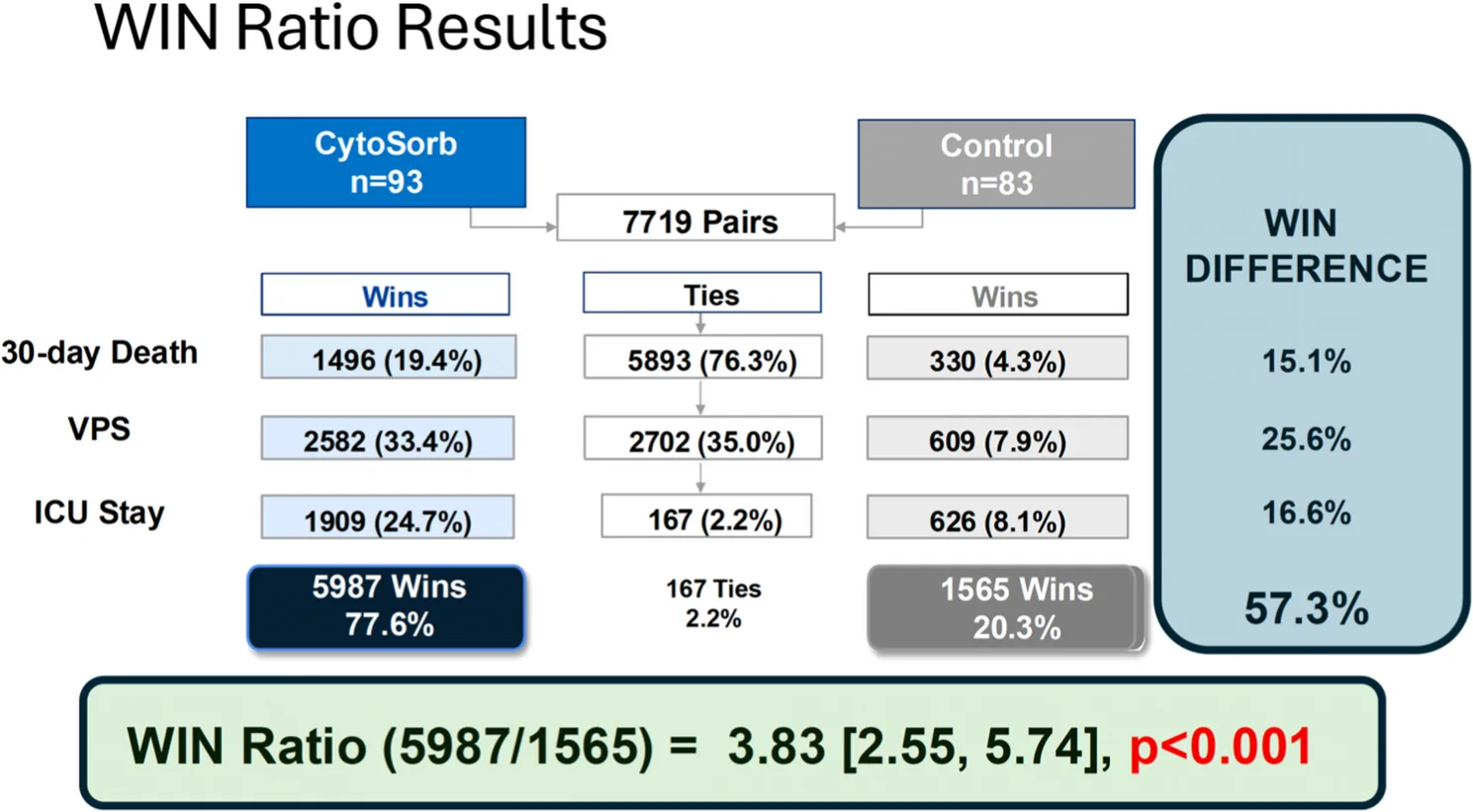
Win ratio results of 7,719 pairs. VPS, Vasoplegic syndrome

The analysis met the primary endpoint in favor of patients receiving intraoperative hemoadsorption (win ratio: 3.83, 95%CI: 2.55, 5.74; *p* < 0.001). A total of 7,713 pairs were analyzed with 5,987 (77.6%) wins in the hemoadsorption group compared to 1,565 (20.3%) wins in controls (167 ties (2.2%) occurred). The win difference was mainly driven by the vasoplegic syndrome accounting for 25.6%, followed by 30-day mortality (15.1%) and ICU stay (16.6%). The win ratio results are illustrated in Fig. 3. Moreover, a separate win ratio analysis including also acute kidney injury into the hierarchical model (4 level analysis) is given in the Supplement.

Multivariate regression analyses were performed for independent predictors of postoperative vasoplegia and 30-day mortality. CytoSorb treatment remained independently associated with a lower risk of postoperative vasoplegia (OR 0.357, 95% CI 0.187–0.682; *p* = 0.0018), while BMI was not significantly associated with vasoplegia (*p* = 0.5998) (Supplemental Table S1). Moreover, multivariate analysis showed that age (*p* = 0.87), sex (*p* = 0.79), EuroScore-II (*p* = 0.74), and preoperative vasopressors (*p* = 0.72) have no impact on postoperative vasoplegia (Supplemental Table S2). The multivariate results impacting 30-day moratlity are presented in Supplemental Table S3.

Mechanical circulatory support with either ECMO or IABP was required in four patients in the hemoadsorption group and six in the control group, *p* = 0.52. Postoperative renal failure requiring hemodialysis occurred in 34 patients. The incidence of new renal failure was more pronounced in the control group (25.3%) compared to the hemoadsorption group (14.0%) without reaching statistical significance (*p* = 0.08). Respiratory failure requiring reintubation occurred in 20 patients, 7 in the hemoadsorption group and 5 in the control group, *p* = 0.77. Postoperative surgical revision was comparable between both groups (*p* = 0.83) (Table 3). There were no significant differences in hospital stay between the groups, however, the win ration was in favor for the hemoadsorption group (24.7% vs. 8.1% wins).

## Discussion

The present comparative study analyzed the clinical effects of intraoperative hemoadsorption in high-risk patients with infective endocarditis undergoing cardiac surgery. The main observations of this report are that in high-risk cardiac surgery patients presenting with infective endocarditis, the intraoperative use of hemoadsorption was associated with a significantly lower incidence of postoperative vasoplegic syndrome, resulting in a lower overall 30-day mortality. Notably, the primary composite endpoint using an unmatched hierarchical win ratio was met in favor of intraoperative hemoadsorption.

The development of postoperative sepsis or vasoplegic syndrome remains an important cause of morbidity and mortality in patients undergoing cardiac surgery for acute infective endocarditis [4]. The reported incidence of vasoplegia after cardiac surgery for infective endocarditis varies considerably due to differences in patient characteristics and perioperative management but remains still high. Nevertheless, the association between vasoplegia and poor results, including mortality, is persistent. Vasoplegic syndrome in IE patients is based on the inflammatory status of the patient, especially in those with “hot” endocarditis, and is exacerbated by the need for intraoperative extracorporeal circulation. The vasoplegic syndrome is characterized by low systemic arterial blood pressure, decreased systemic vascular resistance with near normal or increased cardiac output, requiring high doses of vasopressors [4]. This systemic inflammatory response is orchestrated in large part by high levels of circulating cytokines, a phenomenon referred to in the most severe cases as a “cytokine storm” [5, 11]. Extracorporeal hemoadsorption is a blood purification technology with proven adsorption capacity for cytokines, chemokines, bilirubin, myoglobin, plasma free hemoglobin, and various pharmacological agents such as ticagrelor or rivaroxaban [11–16]. Hemoadsorption therapy has been shown to reduce circulating cytokines at various levels. Therefore, it is plausible that cytokine removal by (intraoperative) hemoadsorption may be a possible preventive measure against postoperative vasoplegia in IE patients undergoing cardiac surgery.

The adjunctive intraoperative use of hemoadsorption in IE has evolved over time from an initial case series to observational studies, to more widespread use, and to evaluation in three randomized controlled trials (RCTs) [17–19]. Many studies have shown a reduced rate of sepsis and/or sepsis-associated mortality and, most importantly, a significant reduction in vasopressor therapy, such as the RCT by Holmén et al. [17]. The REMOVE (Revealing Mechanisms and Investigating Efficacy of Hemoadsorption for Prevention of Vasodilatory Shock in Cardiac Surgery Patients With Infective Endocarditis) trial showed rather neutral results in regard to mortality and the primary outcome (change in sequential organ failure assessment score [ΔSOFA]) [18]. However, the REMOVE trial showed a significant reduction in inflammatory parameters (although not significantly elevated preoperatively) such as interleukin (IL)-6 or Mid-regional pro-adrenomodullin (MR-proADM). Interestingly, in a landmark study by Piskovatska et al., the polymer beads recovered from some of the REMOVE adsorbers were later analyzed: in an in-vitro model, the detrimental effects of an eluate derived from the stored adsorbers on the viability of human aortic endothelial cells were studied [20].

Importantly, previous studies, including the pseudo-randomized data by Metha et al. [21], have clearly demonstrated effective cytokine removal by CytoSorb. However, treatment effects appear to be most pronounced in patients with markedly elevated baseline cytokine levels and a substantial inflammatory burden, which may explain differences between studies [22].

The currently published results remain controversial in clarifying the clinical utility of intraoperative hemoadsorption in IE patients in terms of postoperative morbidity and mortality. A recently published meta-analysis summarizing the currently available data on intraoperative hemoadsorption in IE demonstrated a significant reduction in postoperative vasoconstrictor requirements and a reduction in mechanical ventilation time in patients treated with intraoperative hemoadsorption [23]. The authors also showed an insignificant reduction in perioperative mortality (*p* = 0.05). Of note, a meta-analysis published by Naruka et al. clearly demonstrated a mortality benefit for non-elective cardiac surgery patients with intraoperative hemoadsorption use compared to “normal” low-risk and elective cases [24].

The present study aimed to analyse the clinical effects of intraoperative hemoadsorption in high-risk infective endocarditis (IE) patients in a large single-centre evaluation. It is important to note that, in the current analysis, there were no significant differences between the two groups in terms of their preoperative profile or demographics. Given the balanced baseline characteristics, we decided to skip adjustments such as propensity score matching, with the only difference being the time interval of surgery.

In that regard, the analysis was based on two consecutive time periods before and after the implementation of hemoadsorption. Although the surgical and anesthesiology teams remained unchanged and no major changes in institutional protocols occurred during the study period, the influence of temporal trends cannot be completely excluded. Improvements in perioperative management, postoperative ICU care, or increasing institutional experience may have contributed to the observed outcomes independently of hemoadsorption. Therefore, the observed associations should be interpreted with caution, as unmeasured confounding related to secular changes in clinical practice may have influenced the results.

The preoperatively calculated EuroSCORE-II was high indicating a high-risk cohort of patients undergoing surgery for IE. We decided to use a composite primary outcome using the win ratio method. The win ratio is an emerging alternative for reporting composite outcomes that prioritizes clinically significant events such as mortality while incorporating surrogate measures. The win ratio method compares each subject in the treatment group to each subject in the control group.

The rationale for including 30-day mortality, incidence of vasoplegic syndrome, and ICU stay was based on the currently available literature. Haidari et al. showed a reduction in 30-day and 90-day mortality in IE patients with intraoperative *staphylococcus aureus* hemoadsorption [25]. As mentioned above, some studies, including an RCT by Holmén et al. showed a reduction in postoperative vasopressor requirements [17], and Träger et al. [26] and Rao et al. [27] confirmed a reduced ICU stay when intraoperative hemoadsorption was used in IE patients. In addition, a recently published RCT in 19 versus 19 patients analyzed the effect of intraoperative hemoadsorption in patients with an expected CPB duration of > 90 min [28]. Hohn et al. showed a significantly increased hemodynamic stability in the treatment group (cardiac index 3.2 ± 0.7 vs. 2.47 ± 0.47 L/min/m^2^, *p* = 0.012). The authors also showed a shorter duration of renal replacement therapy in the hemoadsorption group (control group 5.3 days vs. treatment group 2.3 days, *p* = 0.029) [28].

In the current analysis, the primary endpoint was met in favor of intraoperative use of hemoadsorption in high-risk patients undergoing cardiac surgery for IE. Interestingly, the win difference in favor of intraoperative treatment was mainly based on the contribution of postoperative vasoplegia, resulting in a win difference of 25.6%. This lower incidence of postoperative vasoplegia in the hemoadsorption group (24.7% vs. 49.4%, *p* < 0.001) may be the reason for improved morbidity and mortality in the hemoadsorption group. Of note, the present win ratio resulted in a relatively low number of ties, which may be another signal that strengthens the results. However, it should be recognized that the removal of circulating cytokines only affects the inflammatory response. Although postoperative sepsis and vasoplegic syndrome are important causes of postoperative mortality, mortality from other causes cannot be reduced by hemoadsorption therapy. In terms of postoperative renal replacement therapy, postoperative dialysis was found to be comparable with intraoperative hemoadsorption in the present analysis, albeit not significantly (14% vs. 25.3%, *p* = 0.08). Moreover, as hemoadsorption is thought to exert its effects through the removal of inflammatory mediators and cytokines, no measurements of cytokine levels or other inflammatory biomarkers were performed in the present study. Consequently, the observed clinical improvements cannot be directly attributed to cytokine removal, and the proposed mechanism remains speculative. Our findings therefore demonstrate clinical associations rather than a causal relationship between hemoadsorption-mediated cytokine clearance and improved outcomes.

It should be noted that the present study exclusively evaluated the CytoSorb hemoadsorption device. Therefore, the findings cannot be directly extrapolated to other extracorporeal hemoadsorption platforms, such as the oXiris membrane or the Jafron HA series, as these devices differ in their adsorption properties, mechanisms of action, and intended clinical applications [29].

Finally, the addition of intraoperative hemoadsorption incurs additional costs. However, Rao et al. have demonstrated that reduced morbidity and a shorter stay in the intensive care unit (ICU) with hemoadsorption could have significant economic benefits [27]. In the current analysis, we also observed potential benefits regarding postoperative dialysis and ICU stay. Hübner et al. recently analyzed the cost-effectiveness and length of stay of hemoadsorption therapy in ICU patients with septic shock. The authors demonstrated that hemoadsorption was performed at a high-volume Swiss tertiary care center without increasing treatment costs or nursing workload [30]. Another study by Rao et al. demonstrated potential economic benefits associated with reduced ICU length of stay, leading to more efficient resource utilization and lower healthcare costs, even in the base-case scenario without reimbursement for hemoadsorption therapy [31].

### Limitations

There are several important limitations to our study that must be considered when interpreting the results. The current dataset was derived from a single center, the results may not be generalizable to other centers. On the other hand, the results reported in this manuscript may not be directly transferable to smaller institutions with lower case volumes and less specialized infrastructure without a dedicated local endocarditis team. Furthermore, despite being one of the largest analyses to date, the sample size is relatively small. However, due to the balanced preoperative demographics, significant bias is unlikely, so we decided against using a matching method. It should be noted that crude comparisons and the win ratio method may ignore confounding factors. The different inclusion times may also have influenced the results. Therefore, selection bias may have been introduced if unseen confounders remain present. Of note, diagnosis of vasoplegic syndrome was based on predefined clinical criteria rather than comprehensive haemodynamic measurements, and misclassification of patients with other causes of vasopressor requirement cannot be excluded. Moreover, several important variables—including intraoperative and postoperative fluid balance, weight-adjusted vasopressor and inotrope doses, and ventricular function—could not be reported because they were not collected in a standardized manner. Furthermore, standardized measurements of cardiac output/cardiac index (CO/CI) and systemic vascular resistance (SVR) were not available, and the diagnosis of vasoplegic syndrome relied solely on clinical criteria. These limitations increase the risk of misclassification of vasoplegic syndrome, which constitutes the primary study outcome.

Future randomized studies should focus on selecting patients who will benefit from intraoperative hemoadsorption. In addition, this study did not evaluate the continuation of therapy after surgery. This adjunctive concept may benefit patients with IE who develop perioperative renal failure alongside severe hemoadsorption instability and high-grade intraoperative findings. It is important to note that cytokine levels and other inflammatory biomarkers were not assessed, as the study focused on clinically relevant outcome parameters. As a result, the study demonstrates clinical associations only and does not provide mechanistic evidence that cytokine removal was responsible for the observed benefits. Therefore, definitive conclusions regarding the effectiveness of cytokine elimination by the device cannot be drawn. However, these results can be contextualized within the existing literature, such as that of Jansen et al. [11] and Diab et al. [18]. Further research is required to validate the promising results presented in this report and to better define the efficacy of intraoperative hemoadsorption in cases of infective endocarditis.

## Conclusion

In conclusion, intraoperative hemoadsorption appears to reduce the severity of postoperative vasoplegic syndrome and may be associated with reduced morbidity and mortality in selected high-risk patients undergoing surgical treatment for infective endocarditis. However, these findings should be interpreted with caution in light of the methodological limitations of the present study. Although this analysis represents the largest single-centre study of intraoperative hemoadsorption in patients with infective endocarditis, further prospective randomized studies are required to confirm these findings and to investigate the underlying mechanisms.

## Supplementary Information

Supplementary Material 1.

## Data Availability

Data can be provided upon request to the corresponding author.

## Abbreviations

BMI: Body mass index
CABG: coronary artery bypass grafting
CPB: Cardiopulmonary Bypass
CE: Conformité Européenne
CI: Confidence interval
COPD: Chronic obstructive pulmonary disease
CRP: C-Reactive Protein
ECMO: extracorporeal membrane oxygenation
EuroSCORE-II: European System for Cardiac Operative Risk Evaluation
HA: Hemoadsorption
HAT: Hemoadsorption therapy
HR: Hazard ratio
IABP: Intra-aortic balloon pump
ICU: Intensive Care Unithat
IE: Infective Endocarditis
IL: interleukin
IQR: Interquartile range
LVEF: Left ventricular ejection fraction
MR-proADM: Mid-regional pro-adrenomodullin
NYHA: New York Heart Association
RCT: Randomized controlled trial
RRT: Renal replacement therapy
SD: Standard deviation
SOFA: Sequential organ failure assessment score
STROCSS: Strengthening the Reporting of Cohort, Cross-Sectional and Case-Control Studies in Surgery
TAVI: Transcatheter Aortic Valve
