# Supplementary material for: The impact of intraoperative hemoadsorption during cardiac surgery for infective endocarditis
Source: Intensive Care Med Exp. 2026 Oct 14;14:137. doi: 10.1186/s40635-026-00988-0 (PMC13669272; doi:10.1186/s40635-026-00988-0)
Supplement: Supplementary file 1 [file 40635_2026_988_MOESM1_ESM.docx]

**Supplement to:** The impact of intraoperative hemoadsorption during cardiac surgery for infective endocarditis

4-layer win ratio including kidney failure

Win ratio:

**Table S1:**

| **Risk factor** | **OR and 95% CI** | **P-value** |
| --- | --- | --- |
| CS vs. Control | 0.357 (0.187, 0.682) | 0.0018 |
| BMI (per 1 point increase) | 0.984 (0.926, 1.046) | 0.5998 |
| c-statistics=0.635 | | |

BMI: Body mass index

**Table S2:**

| **Risk factor** | **OR and 95% CI** | **P-value** |
| --- | --- | --- |
| CS vs. Control | 0.290 (0.149, 0.566) | 0.0003 |
| Pre-op intubation (Yes vs. No) | 0.395 (0.098, 1.589) | 0.1912 |
| CPB (per 1 min increase) | 0.977 (0.955, 1.000) | 0.0505 |
| ACC (per 1 min increase) | 1.025 (0.996, 1.056) | 0.0924 |
| c-statistics=0.687 | | |

CS: CytoSorb

**Table S3:**

| **Risk factor** | **OR and 95% CI** | **P-value** |
| --- | --- | --- |
| CS vs. Control | 0.270 (0.086, 0.850) | 0.0253 |
| Age (per 10-yr increase) | 1.290 (0.841, 1.978) | 0.2431 |
| Gender (F vs. M) | 3.319 (1.002, 10.993) | 0.0496 |
| EuroSCORE-II (per 1% increase) | 1.062 (1.022, 1.103) | 0.0022 |
| CPB (per 1 min increase) | 1.006 (0.998, 1.014) | 0.1358 |
| c-statistics=0.848 | | |
